# Evaluation of continuous improvement effect of perioperative nursing quality in Da Vinci robot-assisted laparoscopic radical prostatectomy

**DOI:** 10.1097/MD.0000000000042896

**Published:** 2025-06-13

**Authors:** Minshu Zhou, Haiou Qi

**Affiliations:** a Nursing Department, Sir Run Run Shaw Hospital, School of Medicine, Zhejiang University, Hangzhou, Zhejiang Province, China.

**Keywords:** continuous improvement of nursing quality, Da Vinci robot, laparoscopy, prostate cancer

## Abstract

This study explores the clinical effect of continuous improvement of perioperative nursing quality in patients with Da Vinci robot-assisted laparoscopic radical prostatectomy. A total of 222 patients who received Da Vinci robot-assisted laparoscopic radical prostatectomy in our hospital from January to June 2021 were selected as the control group. From July to December 2021, 259 patients who received Da Vinci robot-assisted laparoscopic radical prostatectomy using the perioperative nursing quality continuous improvement model were selected as the improvement group, and the nursing effects of the 2 groups of patients were compared. The placement time of the patients, the incidence of the second berth and the robotic arm collision with the body in the improvement group were significantly less than those in the control group (*P* < .05); the incidence of short-term postoperative adverse events in the improved group (3.47%) was significantly lower than that in the control group (8.11%) (*x*^2^ = 7.096, *P* = .011). Patients in the improvement group had significantly higher satisfaction with nursing services than the control group, and the difference between the groups was statistically significant (*x*^2^ = 9.845, *P* = .002). The continuous improvement of perioperative nursing quality of Da Vinci robot-assisted laparoscopic radical prostatectomy can not only expose the surgical site to the greatest extent, ensure the smoothness of the operation, but also effectively reduce the incidence of adverse events and improve patient satisfaction, which is worthy of clinical promotion and application.

## 1. Introduction

In clinical practice, prostate cancer is one of the most common malignant tumors of the male urinary system. Under the condition that the patient did not suffer tumor metastasis, radical surgery for prostate cancer (mainly including open, laparoscopic, and robot-assisted surgery) is the most effective treatment strategy.^[1]^ Compared with other surgical strategies, the Da Vinci surgical robots, with their high sensitivity, accuracy and precise imaging, can retain the patient’s nerve and urinary control structure to the maximum extent, which is practical and secure.^[2,3]^ The radical prostatectomy combing Da Vinci surgical robots and laparoscopy is also a treatment strategy with a high clinical application rate.^[4]^ However, from the clinical application perspective, since the Da Vinci surgical robots cannot give feedback to the control force, the mechanical arm system would be locked if a puncture device was installed during the operation. In this case, body position change might lead to skin damage.^[5–7]^ Therefore, the body position management of the patients undergoing robot-assisted laparoscopic radical prostatectomy is of irreplaceable importance for the surgical process and patients’ prognosis. Based on this, this study would take the patients who received Da Vinci robot-assisted laparoscopic radical prostatectomy in our hospital from January to June 2021 as the control group, patients who received Da Vinci robot-assisted laparoscopic radical prostatectomy in the improved mode of body position management from July to December 2021 as the improvement group. And then, the clinical value of intraoperative body position management was explored via statistical comparison.

## 2. Data and methods

### 2.1. Baseline data

A total of 222 patients who received Da Vinci robot-assisted laparoscopic radical prostatectomy in our hospital from January to June 2021 were selected as the control group, whose ages were 49 to 75 years old, and body mass indexes were 18.1 to 29.2 kg/m^2^. A total of 259 patients, aged 45 to 73 years, with body mass indexes of 17.9 to 28.7 kg/m^2^, were admitted from July to December 2021 after adopting the improved body position management model and were taken as the improvement group. There was no statistically significant difference in baseline data between the 2 groups (*P* > .05). See Table 1 for details.

**Table 1:** Comparison of baseline data between 2 groups.

Group	Age ($\bar{x}\pm \;s$, yr)	BMI ($\bar{x}\pm \;s$, kg/m^2^)	Clinical stages [n (%)]			
			T1	T2	T3	
The improvement group (n = 259)	56.91 ± 6.34	23.18 ± 2.25	29 (11.20)	177 (68.34)	53 (20.46)	
The control group (n = 222)	57.28 ± 7.11	22.97 ± 2.09	24 (10.81)	153 (68.92)	45 (20.27)	
Statistical value	*t* = 2.037	*t* = 1.994	*x*^2^ = 2.119			
*P* value	.094	.182	.128			
Group	PSA ($\bar{x}\pm s$, ng/mL)	Prostate volume ($\bar{x}\pm s$, cm^3^)	Gleason score [n (%)]			
			≤6	3 + 4	4 + 3	≥8
The improvement group (n = 259)	18.14 ± 1.27	81.62 ± 10.21	72 (27.80)	73 (28.19)	80 (30.89)	34 (13.12)
The control group (n = 222)	18.34 ± 1.33	80.91 ± 9.88	56 (25.23)	69 (31.08)	75 (33.78)	22 (9.91)
Statistical value	*t* = 1.274	*t* = 2.164	*x*^2^ = 1.043			
*P* value	.197	.164	.249			

BMI = body mass indexes.

### 2.2. Methods

The routine perioperative nursing intervention was carried out in both groups, including preoperative health education, psychological nursing, ward management, and postoperative medication guidance; The control group only carried out routine body position management during the operation. Methods: During the operation, the patient’s body position was adjusted to the supine position with the head lowered and foot raised (Trendelenburg position); that is, separate the patient’s legs, fix the patient’s lower legs with restraint straps, fix the arms on both sides of the body, place shoulder pads on the patient’s shoulders, adjust the operating table in front of the surgical robot berth, and make the patient’s head lowered and foot raised, with an included angle of 30° with the horizontal line.

**Figure 1.**
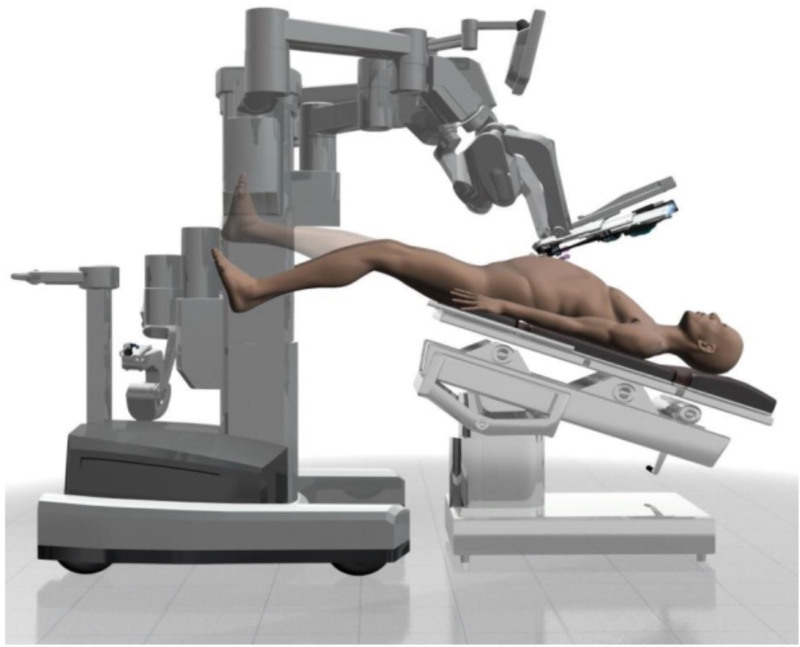
Schematic diagram of Trendelenburg position.

The patients in the improvement group underwent intraoperative body position management. Methods: (1) Preoperative evaluation: The nurses in the operating room should know the patients’ essential information in detail, including limb activity, skin, nutritional status, etc, and evaluate the operation security of the patients. For patients who had been in bed for a long time, were not able to move quickly, had fewer daily activities, or were at risk of forming deep vein thrombosis, pressure ulcers, or causing peripheral nerve damage during the perioperative period, active and effective preventive measures should be taken before the operation. The formation of deep vein thrombosis could be reduced by raising the affected extremity and strengthening the joint activity of the lower limb^[8]^; Inflatable mattress, protective foam pad, or pressure regulating pad could prevent the formation of pressure sore; The potential risk of peripheral nerve injury could be reduced by strengthening the management of body position during operation and placing patients in a functional position; (2) Preoperative health education: Introduce the significance and necessity of intraoperative body position management to patients to improve their cognitive level and ensure the smooth implementation of follow-up medical activities; (3) Body position: Before the patient entered the room, remove the head of the operating table, guide and assist the patient in lying down in a flat position, guide the patient to move his hips to the sacral pad prepared in advance before surgery, and evenly separate his legs; After the completion of anesthesia, the nurses in the operating room should place the patient’s legs on the bed tail plate respectively, horizontally separate them at an angle of about 60°, and then fix the patient’s lower legs with the assistance of a restraint band to prevent displacement during the operation; The upper limbs were wrapped around the body with therapeutic towel, and naturally fixed at the side of the body to keep the Trendelenburg position. The shoulders on both sides are fixed with sponge pads to prevent the body from sliding down due to the Trendelenburg position. After fixing, swing the operating table to an included angle of 30° with the horizontal line, as shown in Figure 1. In positioning, pay attention to keeping warm, respecting patients’ privacy, and avoiding unnecessary skin exposure. (4) Body position adjustment: Adjust the patient’s body position to make the patient’s head lowered and foot raised, and let the lower part of the patient’s knee joint naturally relax as much as possible to reduce the probability of movement due to the discomfort of body position during the operation; Simultaneously, the bedside remote control is used to reduce the bedside by about 30° on the basis of the original height. Finally, with the aid of a protractor, the downward angle of the surgical head and the downward angle of the leg plate are accurately measured to avoid excessive head lowering. (5) Intraoperative protection: During the placement of the Da Vinci surgical robot, the nurse should closely observe and evaluate the security of the body position. In case of abnormal conditions, the angle must be adjusted in time to prevent the machine arm from touching the patient’s body; During the operation, the nurses in the operating room could massage the pressed parts and lower limbs of the patients moderately to prevent the occurrence of deep vein thrombosis and pressure sores.

### 2.3. Evaluation index

(1) The differences in relevant indexes of the 2 groups during the surgical process were compared, including the placement time of body position, the incidence of collisions between arms and arms, and the incidence of secondary berthing. (2) The short-term adverse events of the 2 groups were compared, mainly including deep vein thrombosis, poor restraint, iatrogenic skin injury, peripheral nerve injury, and pressure ulcer. (3) Nursing satisfaction evaluation: The satisfaction of the 2 groups with nursing service was compared by questionnaire, which was divided into 3 levels: complete satisfaction, basically satisfaction, and dissatisfaction; Satisfaction = (complete satisfaction + basically satisfaction) number of people/total number of people × 100%.

### 2.4. Statistical methods

SPSS 20.0 software package was selected for data analysis. Mean ± standard deviation (*x* ± *s*) was used as the expression of measurement data, and the comparison results were tested by *t* test; [n, %] was used as the expression of counting data, and the *x*^2^ test tested the comparison results. Furthermore, *P* < .05 indicates that the difference is statistically significant.

## 3. Results

### 3.1. Comparison of intraoperative indexes between 2 groups

The placement time of body position in the improvement group was lower than that in the control group, and the difference between the groups was statistically significant (*P* < .05); At the same time, the incidence of secondary berthing and the incidence of collisions between mechanical arm and torso, and the difference between the groups was statistically significant (*P* < .05). See Table 2 for details.

**Table 2:** Comparison of intraoperative indexes between 2 groups.

Group	The placement time of body position ($\bar{x}\pm \;s$, min)	The incidence of secondary berthing [n (%)]	The incidence of collisions between mechanical arm and torso [n (%)]
The improvement group (n = 259)	4.10 ± 1.72	6 (2.32)	7 (2.70)
The control group (n = 222)	6.91 ± 2.21	46 (20.72)	19 (8.56)
Statistical value	*t* = 7.330	*x*^2^ = 10.346	*x*^2^ = 5.169
*P* value	<.001	<.001	.009

### 3.2. Comparison of short-term adverse events between 2 groups

The incidence of short-term adverse events in the improvement group (3.47%) was significantly lower than that in the control group (8.11%), with a statistically significant difference between groups (*x*^2^ = 7.096, *P* = .011). See Table 3 for details.

**Table 3:** Comparison of short-term adverse events between 2 groups [n (%)].

Group	Deep vein thrombosis	Poor restraint	Iatrogenic skin injury	Peripheral nerve injury	Pressure ulcer	Total incidence
The improvement group (n = 259)	3 (1.16)	1 (0.39)	0 (0.00)	1 (0.39)	4 (1.54)	9 (3.47)
The control group (n = 222)	4 (1.80)	2 (0.90)	3 (1.35)	2 (0.90)	7 (3.15)	18 (8.11)
*x* ^2^						7.096
*P* value						.011

### 3.3. Comparison of satisfaction with nursing between 2 groups

The questionnaire survey results showed that the satisfaction with nursing in the improvement group was higher than that in the control group, with a statistically significant difference between the groups (*x*^2^ = 9.845, *P* = .002). See Table 4 for details.

**Table 4:** Comparison of satisfaction with nursing between 2 groups [n (%)].

Group	Complete satisfaction	Basically satisfaction	Dissatisfaction	Satisfaction
The improvement group (n = 259)	162 (62.55)	82 (31.66)	15 (5.79)	244 (94.21)
The control group (n = 222)	121 (54.50)	69 (31.08)	32 (14.42)	190 (85.58)
*x* ^2^				9.845
*P* value				.002

## 4. Discussion

As an effective auxiliary means, the application of the Da Vinci surgical robot in laparoscopic radical prostatectomy, on the one hand, improves the accuracy of surgery, which is conducive to the improvement of surgical effect; On the other hand, it effectively alleviates the injury caused by the operation itself and avoids the occurrence of adverse events such as infection.^[9]^ However, from the clinical application perspective, the Da Vinci surgical robot also has some shortcomings.^[10–12]^ For example, the space occupied by the system is ample; Secondly, tactile perception and mechanical force feedback are relatively lacking. If the patient’s position is unreasonable, it is likely for the robot arm to collide with the patient’s body or the base to squeeze the limb, which will not only affect the smoothness of the operation but also have specific potential security hazards.

For a long time, Da Vinci surgical robot-assisted laparoscopic radical prostatectomy has selected the Trendelenburg position as the patient’s body position. This position allows the patient’s intestinal part to be naturally pulled to the head side by gravity, thus far away from pelvic organs, which has significant advantages in pelvic organ surgery via the abdominal approach. However, when adopting the Trendelenburg position (the position of head lowered and foot raised), the patient is prone to complications such as eyelid edema and face and neck congestion, which will increase the patient’s discomfort and affect the surgical process and security.^[13]^ On the other hand, after the Da Vinci surgical robot is placed, the operating table and the patient’s position cannot be adjusted again. Once the body position is unreasonable during the operation, the surgical instruments must be removed entirely, which will significantly prolong the operation time and increase the patient’s exposure time during the operation; In surgery, the prolongation of patients’ operation time means the increase of blood loss and risk of various complications^[4]^; Therefore, in the Da Vinci robot-assisted laparoscopic radical prostatectomy, the implementation of body position management has considerable clinical significance for the surgical process and security. From the data comparison of the results of this study, the placement time of the patients, the incidence of the second berth and the robotic arm collision with the body in the improvement group were significantly less than those in the control group (*P* < .05), which were consistent with the previous research conclusions of relevant scholars.^[14–16]^

The reasons why the placement time of body position of the improvement group was shorter than that of the control group could be as follows: the nursing staff of the improvement group had thoroughly evaluated the patients’ physical condition, carried out health education before the operation, and paid attention to postural fixation and protective measures, which avoided adjusting the secondary adjustment during the operation, thus saved the placement time of body position; While in the control group, only routine posture management was adopted, and the nursing staff did not conduct preoperative health education to the patients as in the improvement group and did not strengthen the intraoperative protection measures to body posture. Consequently, the patient’s body position might need to be adjusted repeatedly during the operation for various reasons, suggesting the necessity and clinical value of intraoperative posture management from the side.

From the perspective of adverse events, the common adverse events after applying the Da Vinci surgical robot could be summarized as follows: (1) Stress injury: According to the existing literature,^[17,18]^ the longer the operation time, the higher the probability of stress injury. In this study, the operation time of the 2 groups was about 120 minutes, which was within an acceptable range. However, the robot radical prostatectomy usually adopts the Trendelenburg position (head lowered and foot raised), which significantly increases the shearing force between the patient’s skin and the operating table compared with the surgery in the conventional supine position. In addition, with the patient’s center of gravity moving downward, the squeezing force of the patient’s shoulder bracket and shoulder is greatly enhanced. With the aid of a protractor, the surgical head’s downward angle and the leg plate’s downward angle can be accurately measured to avoid excessive head lowering, which can effectively avoid the occurrence of stress injury. (2) Squeeze caused by the mechanical arm: As the Da Vinci surgical robot lacks force feedback and touch response, the movement of the mechanical arm is prone to cause crushing injury. If the angle of the patient’s legs separating is unreasonable or the legs are not flat during the operation, it is easy to cause this adverse event; Through body position management and the adjustment of the angle of the patient’s legs to ensure that the manipulator can operate smoothly between the patient’s legs without affecting the operation, and the adjustment the height of the bedside can effectively avoid the collision of the manipulator. (3) Nerve injury: The position selection of surgery is a passive choice according to the needs of the surgery. In addition, using anesthetic drugs will cause patients to have different levels of sensory disorders; If the body position is placed unreasonably, nerve compression or excessive extension may easily lead to nerve injury. From the data of this study, the incidence of short-term adverse events in the improvement group (3.47%) was significantly lower than that in the control group (8.11%) (*P* < .05), which suggested that body position management could ensure the security and continuity of the operation in Da Vinci surgical robot-assisted laparoscopic radical prostatectomy.

To further analyze the application effect of body position management in the Da Vinci surgical robot-assisted laparoscopic radical prostatectomy, this study also conducted a comparative analysis of the satisfaction with the nursing of the 2 groups. The results showed that the patients in the improvement group were more satisfied with nursing care (94.21%) than the control group (85.58%) (*P* < .005), indicating that intraoperative body position management could improve the patients’ satisfaction towards surgery, which was consistent with the research conclusions of Luo Min et al.^[19]^

Due to the lack of professional medical knowledge, most patients’ evaluation of medical and nursing activities mainly stayed at the subjective level, which contained the following 2 aspects: (1) “Whether the subjective feelings were taken care of,” before the implementation of body position management, the improvement group carried out relevant health education, which improved the patient’s cognitive level and simultaneously let the patients genuinely feel the heartfelt intentions of the medical staff, which had a promoting effect on the establishment of a harmonious doctor–patient relationship; (2) “Whether the treatment and rehabilitation process met the expectations of patients,” which was also the core factor affecting patient satisfaction. Implementing body position management shortened the operation time, reduced the incidence of adverse events during and after the operation, and improved patients’ negative emotions, which were conducive to establishing a harmonious doctor–patient relationship.^[20]^

To sum up, the Da Vinci surgical robot-assisted laparoscopic radical prostatectomy is an advanced means of clinical treatment of prostate cancer, which can improve the accuracy of surgery and reduce surgical trauma. However, due to its limitations, if the patient’s body position was unreasonable during the operation, it would prolong the operation time and lead to the risk of adverse events during and after the operation. Therefore, it is of great clinical significance to cooperate with implementing continuous improvement measures for nursing quality, such as perioperative body position management, to ensure patients’ surgical effect and security.

## Author contributions

**Conceptualization:** Minshu Zhou, Haiou Qi.

**Data curation:** Minshu Zhou, Haiou Qi.

**Formal analysis:** Minshu Zhou, Haiou Qi.

**Investigation:** Minshu Zhou, Haiou Qi.

**Resources:** Minshu Zhou, Haiou Qi.

**Software:** Minshu Zhou, Haiou Qi.

**Supervision:** Haiou Qi.

**Validation:** Haiou Qi.

**Visualization:** Minshu Zhou, Haiou Qi.

**Writing – original draft:** Minshu Zhou, Haiou Qi.

**Writing – review & editing:** Minshu Zhou, Haiou Qi.
